# Exploring the medicinal value of Boletus from the dual perspectives of modern pharmacology and traditional Chinese medicine theory

**DOI:** 10.3389/fphar.2026.1792398

**Published:** 2026-08-24

**Authors:** Yuxin Li, Yuxin Ma, Ting Zhu, Baoyi Peng, Shufang He, Xinyue Qiu, Xin Zhan, Zihan Xing, Yifan Gao

**Affiliations:** 1 Department of Basic Teaching, Zhuhai Campus of Zunyi Medical University, Zhuhai, Guangdong, China; 2 Department of Nursing, Zhuhai Campus of Zunyi Medical University, Zhuhai, Guangdong, China; 3 Department of Pathology, Basic Medical Sciences, Zunyi Medical University, Zunyi, Guizhou, China

**Keywords:** bioactive metabolites, *B*oletus, medicinal mushrooms, pharmacological effects, traditional Chinese medicine (TCM)

## Abstract

The genus *Boletus* is widely distributed in temperate regions and contains diverse bioactive compounds, particularly polysaccharides and flavonoids. *Boletus* has been reported to exhibit antioxidant, anti-inflammatory, anticancer, gut microbiota-modulating, antihyperglycemic, and muscle-protective activities, mainly due to its polysaccharides, phenolic compounds, and key minerals such as selenium and zinc. In Traditional Chinese Medicine (TCM), *Boletus* is described as having a slightly sour and spicy taste, as well as a neutral nature, and it is believed to affect the heart and spleen meridians. In addition to clearing heat and relieving vexation, it is known to nourish blood and tonify qi, circulate blood and relax tendons, and remove wind and transform dampness. This review examines the pharmacological value of *Boletus* from the perspectives of both modern pharmacology and TCM theory, highlighting the intersection between its contemporary pharmacological actions and traditional therapeutic effects. Moreover, we creatively suggest a path of self-synergy and mutual synergy for *Boletus*’s application, and provide new insights for its modernization in TCM and interventions for chronic diseases.

## Graphical Abstract

**Figure:**
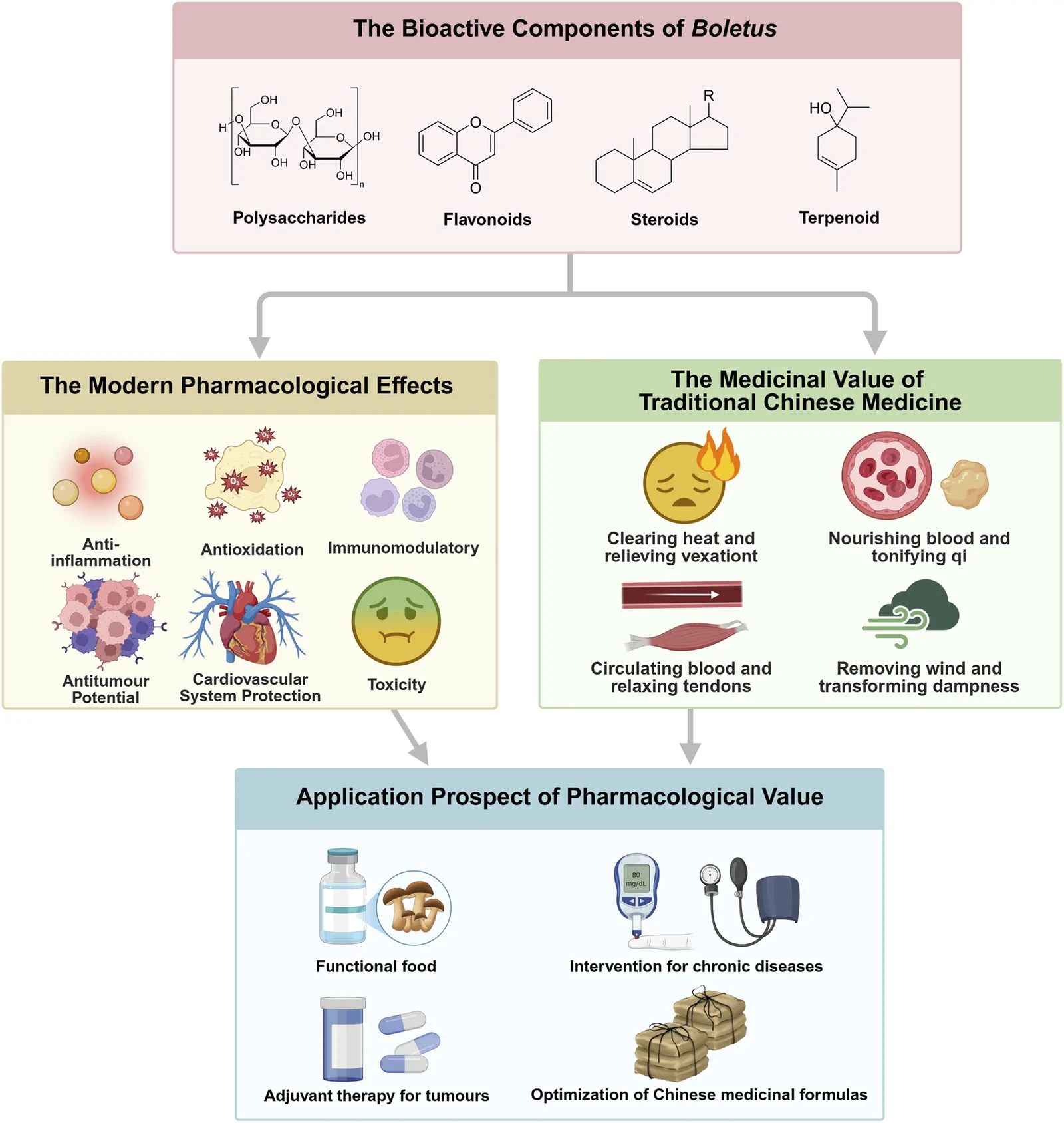
Created in BioRender. gN, <í. (2026) https://BioRender.com/h801okj.

## 1 Introduction

The genus *Boletus belongs* to the phylum Basidiomycota, class Agaricomycetes, order Boletales, and family Boletaceae. It includes a wide variety of large forest fungi that are common in temperate regions of Asia, Europe, and North America. Over 400 species have been identified worldwide, with 199 edible species distributed across southwestern China (Taihui and Bin, 2002). In addition to being loved by foodies all over the world for its unique taste and substantial nutritional value, *Boletus* also attracts a lot of attention in the medical community because of its wide range of bioactive compounds and exceptional therapeutic potential.

In this review, *Boletus* refers to the genus as a whole. However, because different species within the genus may vary in chemical composition and biological activity, representative species are discussed throughout the manuscript where appropriate.

In Eastern and Western traditions, mushrooms have been regarded as symbols of mystery and wellness, respectively. This cultural contrast offers a compelling starting point for scientific inquiry. Therefore, this article aims to explore the therapeutic potential of *Boletus* by integrating the conceptual framework of Traditional Chinese Medicine (TCM) with contemporary pharmacological evidence. The ultimate goal is to provide a solid theoretical foundation for further research and application, promoting the broader utilization of *Boletus* resources.

To facilitate understanding for readers unfamiliar with Traditional Chinese Medicine (TCM), the concepts used in this review should be regarded as part of a traditional theoretical framework rather than a direct biomedical taxonomy. Accordingly, terms such as clearing heat, nourishing blood, tonifying qi, promoting blood circulation, and dispelling dampness are used here to describe the holistic therapeutic rationale of *Boletus*, which may be interpreted in modern scientific language as involving mechanisms associated with inflammation, oxidative stress, immune modulation, microcirculation, and tissue repair.

**FIGURE 1:**
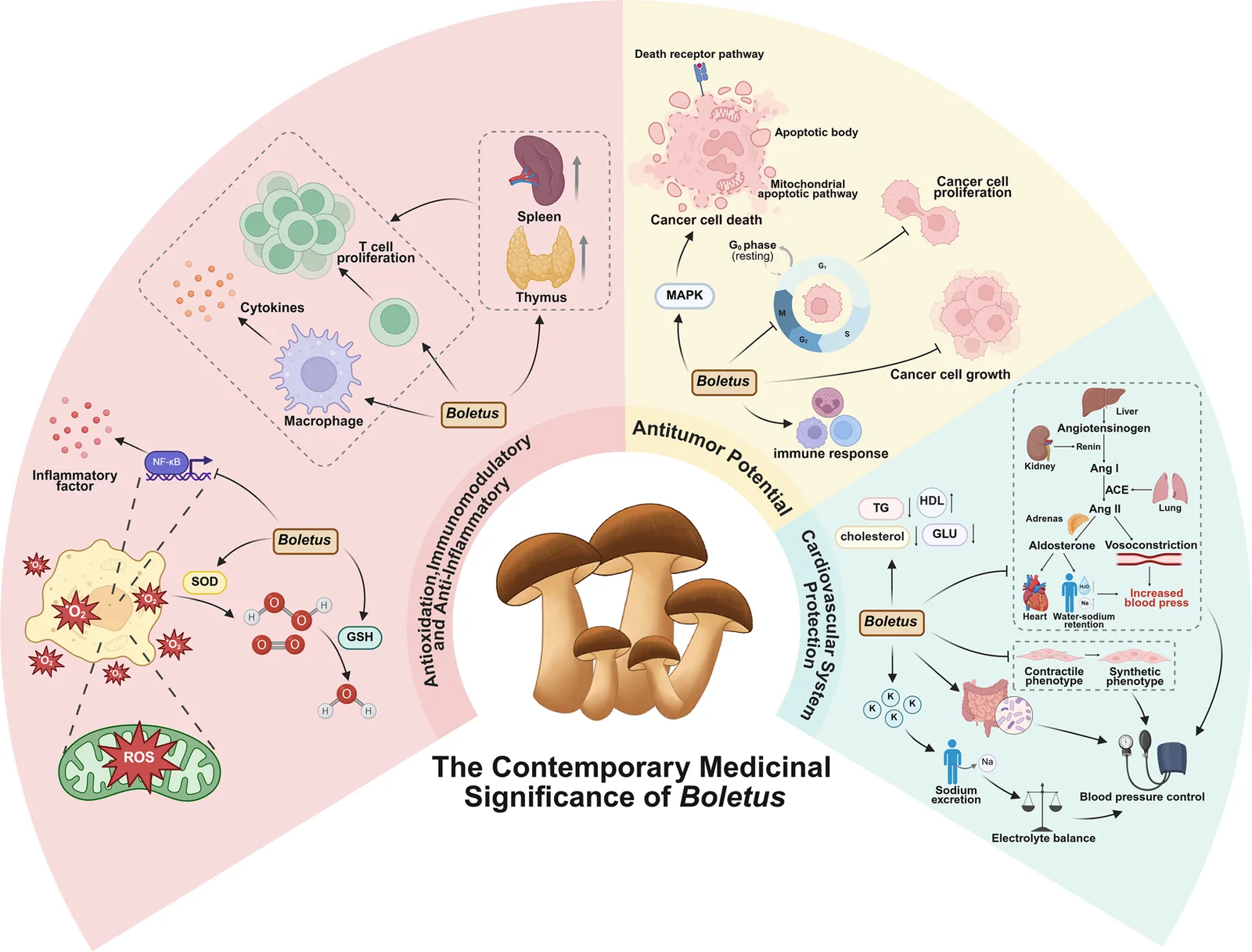
The Contemporary Medicinal Significance of *Boletus*. This schematic summarizes the major pharmacological activities and potential mechanisms of Boletus-derived bioactive compounds. *Boletus* exhibits antioxidant, anti-inflammatory, and immunomodulatory effects by reducing reactive oxygen species (ROS), enhancing antioxidant defenses such as SOD and GSH, regulating inflammatory cytokine production, modulating macrophage activity, and influencing T-cell proliferation and immune organ function. *Boletus* shows potential antitumor activity by regulating cancer-related signaling pathways, promoting cancer cell apoptosis, inhibiting cancer cell proliferation and growth, and modulating antitumor immune responses. *Boletus* also displays cardiovascular and metabolic protective effects, including regulation of lipid metabolism, blood glucose balance, blood pressure control, angiotensin-converting enzyme-related pathways, and vascular function. Together, these activities highlight the broad therapeutic relevance of *Boletus* in oxidative stress, inflammation, immune dysfunction, tumor progression, and cardiovascular-metabolic disorders. Created in BioRender. gN, <í. (2026) https://BioRender.com/h801okj.

**FIGURE 2:**
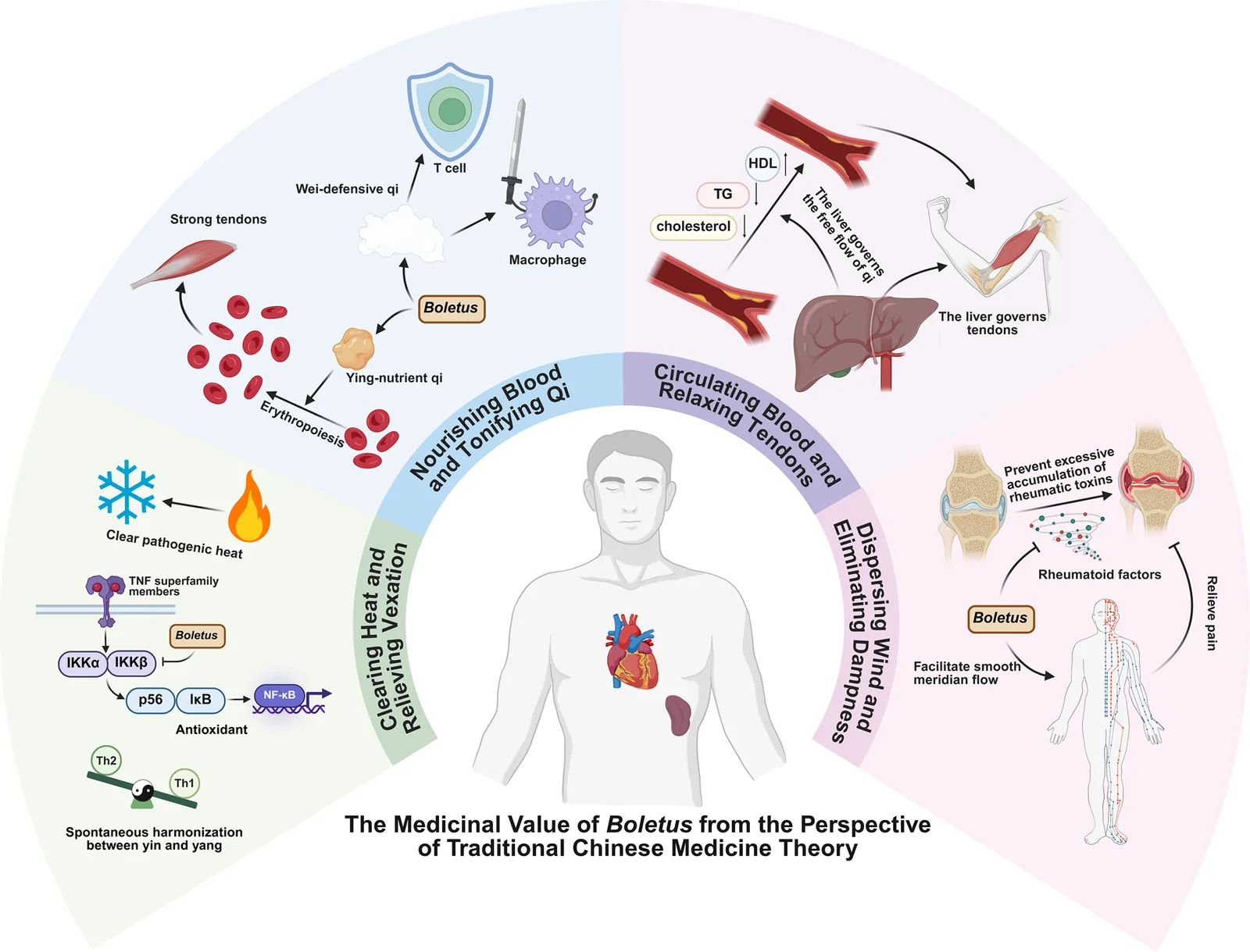
The medicinal value of *Boletus* from the perspective of Traditional Chinese Medicine (TCM) theory. This schematic summarizes the major traditional functions of *Boletus* and their possible modern biological interpretations. Arranged clockwise from the upper left, the four sections illustrate nourishing blood and tonifying qi through erythropoiesis and immune regulation; circulating blood and relaxing tendons through the regulation of lipid metabolism and vascular function; dispelling wind and eliminating dampness through the alleviation of rheumatic symptoms and support of joint health; and clearing heat and relieving vexation through antioxidant, anti-inflammatory, and immune-regulatory activities. These proposed relationships should be regarded as conceptual interpretations rather than direct biomedical validation of TCM theory. Created in BioRender. gN, <í. (2026) https://BioRender.com/h801okj.

Recent research shows that species within the genus *Boletus* are rich in polysaccharides, terpenoids, and numerous other bioactive compounds, with more than 217 compounds reported, although their exact compositions vary among species. Accumulating preclinical evidence indicates that these constituents exhibit antioxidant, anti-inflammatory, immunomodulatory, antitumor, hypoglycemic, hypolipidemic, and cardiovascular protective activities through multi-component and multi-target mechanisms. Historical TCM documents such as *Dian Nan Ben Cao* record the ability of *Boletus* to nourish the “Five zang”(五脏) organs. It is reported that it is used to clear heat, relieve vexation, nourish blood, and treat gastrointestinal discomfort and stomach pain (Mao and Guo-You, 2013). However, there is still work to be done in combining the insights of TCM with the pharmacology of *Boletus* and fully understanding the relationship between its active ingredients and therapeutic effects. The contemporary pharmacological significance of *Boletus* and the proposed conceptual relationships between TCM concepts and modern pharmacological observations are summarized in Figures 1, 2, respectively.

The literature included in this review was retrieved from PubMed, Web of Science, and Google Scholar up to May 2026 using combinations of keywords related to *Boletus*, phytochemistry, pharmacological activities, and Traditional Chinese Medicine (TCM). Additional relevant studies were identified through manual screening of reference lists, and only publications directly related to the medicinal, phytochemical, pharmacological, and ethnopharmacological properties of *Boletus* were included.

## 2 Systematic analysis of the bioactive compounds in *Boletus*

### 2.1 Polysaccharides

Polysaccharides are considered to be one of the key biologically active components in *Boletus*, which is composed of more than a dozen monosaccharides connected by glycosidic bonds. They show antioxidant, antitumor, anti-aging, and immunomodulatory properties. The polysaccharides in *Boletus* are mainly composed of β-(1→3)-D-glucans, and polysaccharides with higher branches show stronger immunomodulatory activity (Xiao et al., 2020). Although the clear relationship between molecular weight and antioxidant capacity has not been determined, factors such as conformation and glycosidic bond type simultaneously affect antioxidant properties. For example, β-configured polysaccharides are more likely to form triple-helix structures related to antioxidant activity. The higher the proportion of β-configured polysaccharides, the stronger the antitumor and immunomodulatory effects (Tian et al., 2025). Studies have shown that the increase of sulfate groups in small molecular polysaccharides with pyranose rings is associated with strong antioxidant effects. *Boletus* polysaccharides show considerable structural diversity, and their antioxidant activity also varies according to different cotyledon sites. For example, the DPPH free radical scavenging activity in cap polysaccharides contrasts sharply with the DPPH free radical scavenging activity in the stipule polysaccharides (Zhang et al., 2018).

A comparative analysis showed that the rate of *Boletus aereus* polysaccharides by the ultrasonic-assisted composite enzyme method (18.47%) is better than that of the traditional hot water extraction method (5.64%) and ultrasound extraction method (6.74%) (Jun-jie et al., 2024). The antioxidant capacity is positively correlated with both polysaccharide concentration and alcohol precipitation concentration, with optimal antioxidant activity observed at an ethanol concentration of 80%.

### 2.2 Flavonoids

Flavonoids, classified as polyphenolic compounds, are secondary metabolites produced during plants’ metabolism. They are widely found in numerous mushrooms and exhibit various pharmacological activities such as antioxidant, anti-inflammatory, antiviral, antitumor, hypoglycemic, etc. Flavonoid compounds in *Boletus* have rich chemical diversity. It was found that the purified *Boletus* flavonoids (B-Fla) contained 20 kinds of flavonoids, and the purified Fla removal rate of DPPH free radicals can reach 86.59%. The cleaning power is close to that of vitamin C, and it has excellent antioxidant capacity (Yue, 2024). Flavonoids inhibit oxidative stress reactions by activating AP-1 (Activator Protein-1) and NF-κB (Nuclear Factor kappa-light-chain-enhancer of activated B cells), removing free radicals by hydrogen ion transfer mechanisms, improving the activity of antioxidant enzymes, and inhibiting the enzymes responsible for free radical generation. This ultimately lessens oxidative stress-induced damage to cells, showcasing significant antioxidant properties. From the level of antioxidant mechanisms of polyphenols, it can simultaneously produce two mechanisms of providing hydrogen ions and transferring its own electrons, thus converting intracellular free radicals into stable substances to reflect the antioxidant capacity (El-Saadony et al., 2024).

The commonly used extraction method for flavonoids from *Boletus* is ultrasonic-assisted ethanol extraction, which is efficient, cost-effective, and simple to perform. Guo Lei developed a mathematical model using response surface methodology to optimize extraction parameters, thereby enhancing flavonoid yield (Lei et al., 2020b). In terms of the purification process of flavonoids using macroporous resins, the purified *Boletus* edulis extracts obtained with D101 resin (49.77%) and NKA-9 resin (32.18%) showed stronger antioxidant capacities than the crude extract (Lei et al., 2020a).

### 2.3 Steroidal compounds

The steroidal compounds found in *Boletus* primarily include ergosterol and its derivatives, which are a class of saturated or unsaturated secondary alcohols, with ergosterol exhibiting a precise scavenging effect on DPPH free radicals, thus demonstrating antioxidant potential. The steroidal compounds in *Boletus* also exhibit certain antibacterial, antitumor, and antiviral activities (Wei, 2010). There are variations in the types and amounts of steroidal compounds among different species of *Boletus*. For example, research has found several steroidal compounds, including ergosterol-7,22-diene-3β,5a,6β-triol, as well as ergosterol peroxides, can be isolated from the *Leccinum extremiorientale* (Jin-Ming et al., 2003). In the *Retiboletus kauffmanii*, ergosterol, β-sitosterol, and 5α,8α-ergosterol peroxide have been isolated (Lin et al., 2017). Currently, the extraction of steroidal compounds from *Boletus* employs solvent extraction methods using organic solvents such as ethanol and methanol, followed by chromatographic separation and purification (Bingji et al., 2007).

### 2.4 Terpenoids

Terpenoids, a class of natural compounds formed by various linkages of oprene or isoprene; they exhibit antioxidant, anti-inflammatory, and antitumor effects, are widely found in nature, and are active components in many traditional Chinese medicines. Edible fungi contain a substantial amount of triterpenoids and sesquiterpenoids, with diterpenes and sesquiterpenes being the predominant structures in *Boletus*, mostly consisting of linear sesquiterpenes (Yang, 2018). At present, the research on terpenoids of *Boletus* mainly focuses on the extraction and inducers of synthesis of triterpenoids, while the research on their biological activities and mechanisms of action is relatively limited. Triterpenoids in edible fungi are famous for their liver protection, lipid-lowering, anti-inflammatory, and antioxidant properties, and some of them also show antitumor activities (Hongfei and Qing, 2023). The three sesquiterpenoids in *Boletus* calopus showed only weak antibacterial activity against a few pathogens, such as the cucumber wilt disease fungus and wheat scab fungus. (Wei, 2010).

Common methods for extracting terpenoids from fungi include solvent extraction, ultrasonic-assisted extraction, microwave-assisted extraction, and supercritical CO_2_ extraction. Each method has certain shortcomings, and the extraction technology needs to be further improved to achieve wider applications (Yan et al., 2024).

## 3 Modern pharmacological research on *Boletus*

*Boletus* contains diverse bioactive compounds, including polysaccharides, flavonoids, phenolic acids, terpenoids, and sterols, which confer multiple pharmacological activities such as antioxidant, anti-inflammatory, and immunomodulatory effects. The antioxidant effects are primarily mediated by scavenging reactive oxygen species (ROS) and enhancing cellular antioxidant enzymes, including superoxide dismutase (SOD), catalase (CAT), and glutathione (GSH) levels. Anti-inflammatory actions involve downregulation of key inflammatory mediators such as cyclooxygenase-2 (COX-2) and inducible nitric oxide synthase (iNOS) through suppression of the NF-κB signaling pathway. Moreover, immunomodulatory properties are achieved by modulating immune receptors like TLR4/MyD88 and balancing cytokine production, reinforcing host defense, and mitigating inflammatory responses. These effects are further complemented by regulation of signaling pathways, including MAPK and PI3K/AKT, enhancing therapeutic potential in inflammation- and immunity-related diseases.

### 3.1 Antioxidant effects

The antioxidant activity of *Boletus* is mainly attributed to the synergistic effects of multiple bioactive constituents, including polysaccharides, flavonoids, polyphenols, and sterols, which act through different molecular targets and pathways (Jing-Bo et al., 2020). These components remove free radicals by providing hydrogen and transferring electrons, and inhibit the formation of free radicals by chelating metal ions such as Fe^2+^ and competitive binding pathways (Qiu-Ming et al., 2018). Additionally, the antioxidant activity of *Boletus* is reflected in its ability to inhibit lipid peroxidation (LPO), activate intrinsic enzyme systems, regulate gene expression, and restore coenzyme systems (Qiao-Ran et al., 2019).

Polysaccharides are major contributors to this activity. Both crude and purified polysaccharides from wild edible *Boletus* exhibit antioxidant effects, with purified fractions demonstrating superior activity (Li-hong et al., 2008). The polysaccharides of *Boletus auripes* possess strong DPPH-removal, ion-removal abilities, and significant chelating ability (Jing-bo et al., 2020; Yan W. et al., 2021). The steroidal compound ergosterol in *Boletus* can effectively eliminate DPPH free radicals. *Boletus edulis* polysaccharides (BEP) can enhance the body’s antioxidant ability by reducing serum aspartate aminotransferase and alanine aminotransferase activities, as well as hepatic malondialdehyde levels, thereby protecting against liver damage (Qiao-Ran et al., 2019). The crude polyphenolic extract from *Leccinum crocipodium* efficiently removes DPPH· and ABTS+·, reduces Fe^3+^, and strongly chelates Fe^2+^ (Qiu-Ming et al., 2018). Furthermore, *Boletus* can enhance the activity of antioxidant enzymes, such as superoxide dismutase (SOD) and glutathione peroxidase (GSH-Px), thereby exerting a comprehensive antioxidant effect (Kuaifen et al., 2024).

Beyond these direct radical-scavenging and enzyme-modulating effects, the antioxidant activity of *Boletus* exhibits notable structural dependence and extraction-related variation. Polysaccharides from the cap and stipe of the same species show contrasting DPPH scavenging activities, indicating that tissue-specific differences in polysaccharide composition affect potency (Zhang et al., 2018). Moreover, polysaccharides with a higher proportion of β-configured residues, which favor triple-helix conformations, demonstrate stronger antioxidant capacity (Tian et al., 2025). Regarding extraction methods, ultrasonic-assisted composite enzyme processing yields polysaccharide fractions with superior antioxidant activity compared to conventional hot water extraction, and the optimal ethanol concentration for precipitation is 80%, where the antioxidant capacity is maximized (Jun-jie et al., 2024). Purification of flavonoid-enriched extracts using macroporous resins further enhances their antioxidant potential relative to crude extracts (Lei et al., 2020a). These findings highlight that both molecular structural features and manufacturing conditions critically influence the final antioxidant efficacy.

In addition, accumulating *in vivo* evidence further supports the hepatoprotective potential of *Boletus*. *Boletus aereus* polysaccharides mitigate alcohol-induced liver injury by modulating the oxidative stress-mediated NF-κB pathway (Zhang et al., 2020). In a streptozotocin-induced type 2 diabetes mellitus rat model, *Boletus* polysaccharides lowered fasting blood glucose, triglyceride, and malondialdehyde levels while improving hepatic histopathology, suggesting a protective role against diabetic hepatopathy (Xiao et al., 2019). These observations confirm that the antioxidant actions of *Boletus* are not merely biochemical phenomena but are directly relevant to disease prevention and organ protection in multiple pathological contexts.

From the perspective of TCM, the robust and multifaceted antioxidant activity of *Boletus* provides a modern scientific rationale for its traditional use in “clearing heat and relieving vexation” (清热解烦). Oxidative stress is a key driver of inflammatory, febrile, and heat-related pathologies. By neutralizing free radicals, chelating pro-oxidant metal ions, and boosting endogenous antioxidant enzymes, *Boletus* directly counteracts the pathogenic “heat” arising from Yin-Yang imbalance, thereby aligning with the TCM goal of eliminating excess pathogenic heat and restoring physiological harmony.

### 3.2 Immunomodulatory and anti-inflammatory effects

Flavonoids in *Boletus* can reduce inflammation by blocking the NF-κB signaling pathway and reducing the release of inflammatory factors (El-Saadony et al., 2024). The ethanol extract of *Boletus impolitus Fr.* inhibits nitric oxide (NO) production, indicating its anti-inflammatory potential (Taofiq et al., 2016). In the asthma mouse model, BEP can effectively increase the anti-inflammatory factor interleukin-4 (IL-4) and reduce interferon-gamma (IFN-γ), thereby enhancing the anti-inflammatory response (Wu et al., 2016). Chang Guofei et al. found that the bioactive compounds in extracts of *Boletus edulis* exert anti-inflammatory effects by influencing several inflammation-related signaling pathways, including the NF-κB pathway, thereby alleviating inflammatory bowel disease (IBD) in mice (Guofei et al., 2025). Therefore, *Boletus* has the potential to slow down the development of the disease by inhibiting inflammatory reactions. The antioxidant effect of the active components in *Boletus* is closely related to the anti-inflammatory effect. These effects can directly protect cells and tissues from oxidative stress and inflammation, and also provide a favorable cellular microenvironment for the normal function and antitumor activity of the immune system. Among them, the action mode of multi-component and multi-target is highly consistent with the connotation of “clearing heat and relieving vexation” in TCM.

Collectively, current evidence suggests that the anti-inflammatory effects of boletes are not attributable to a single constituent but rather to the combined actions of polysaccharides, phenolic compounds, sterols, and other small molecules acting through pathways such as NF-κB/MAPK-related inflammatory signaling. These components may act through free-radical scavenging, metal-ion chelation, enhancement of endogenous antioxidant enzymes, inhibition of lipid peroxidation, and regulation of NF-κB/MAPK. This multi-component and multi-pathway pattern provides a pharmacological explanation for the traditional function of “clearing heat and relieving vexation,” although direct clinical evidence remains limited.

Building on these anti-inflammatory actions, *Boletus* also exerts potent immunomodulatory effects, which are closely intertwined with its regulation of inflammation. In *Boletus* species, polysaccharide components are often reported as the main contributors to immunomodulatory activity. Polysaccharides extracted through various methods can stimulate antibody production and enhance the performance of immune cells, thus fortifying the body’s immunity. This immunomodulatory function is also reflected in enhancing the function of immune organs, improving immune damage, and providing essential nutrition.

The polysaccharides BSF-X and BSF-A isolated from *Xiaojin Boletus speciosus Frost* have strong immune-modulating effects. BSF-X can stimulate T cell proliferation and promote cells from the G0/G1 checkpoint into the G2/M and S phases. It can also upregulate the expression of inflammatory genes and activate macrophages to secrete pro-inflammatory cytokines such as interleukin-6 (IL-6) and tumor necrosis factor-α (TNF-α) (Zhu et al., 2019). Similarly, BSF-A and water-soluble polysaccharide LRP-1 from *Leccinum rugosiceps* can also activate macrophages and other immune-effector cells, enhancing the host’s ability to detect and destroy tumor cells (Hou et al., 2014; Li et al., 2020). BEP can be obtained by alcohol precipitation after water extraction, which can significantly increase the weight of immune organs (such as thymus and spleen), boost the peripheral blood white-cell counts, and significantly promote the transformation of spleen lymphocytes (Wei and Xincheng, 1999). Wild *Boletus* has a significant effect on improving the cellular, humoral, and non-specific immune function of mice caused by the immunosuppressive drug cyclophosphamide (CP) (Jia et al., 2007). Su Lu et al. discovered that serum containing a certain concentration of *Lanmaoa asiatica* significantly promotes the proliferation of splenic lymphocytes in mice (Lu et al., 2018). Moreover, they both demonstrated that *Boletus* has a pronounced enhancing effect on T cell-mediated cellular immunity. In addition to the rich nutrients found in *Boletus*, the various mineral elements it contains, particularly essential elements such as copper, iron, phosphorus, calcium, and manganese, play a crucial role in metabolic activities and the maintenance of normal immune system functions.

The immunomodulatory effects of *Boletus* in mice are also closely related to the extraction methods of *Boletus*. Lipid-based extracts exhibit stronger immunomodulatory effects than water-based extracts, with an obvious effect on immunologic enhancement of female mice compared to male mice (Wang et al., 2012). However, further research is still needed to explore the specific components, their mechanisms, and the relationships between these factors and their efficacy.

In summary, the immunomodulatory and anti-inflammatory effects of *Boletus* are intrinsically linked, forming a core axis of its pharmacological activity. The ability of *Boletus* to regulate immune cell function (e.g., T cells, macrophages) is often accompanied by a parallel modulation of inflammatory mediators (e.g., cytokines, NO). This integrated action directly protects tissues from damage and creates a favorable microenvironment for host defense against tumors and other chronic diseases, aligning with the TCM principle of “strengthening Wei-defensive qi while clearing pathogenic heat (扶正祛邪).”

### 3.3 Antitumor effects

Extracts from *Boletus* primarily activate the caspase cascade via both mitochondrial apoptotic pathway and death receptor pathway, thereby inducing apoptosis in tumor cells. The protein D1 isolated from *Boletus bicolor* is capable of inhibiting the proliferation of non-small cell lung cancer (NSCLC) cell lines (A549 and H1299 cells). It activates the mitogen-activated protein kinase (MAPK) signaling pathway *in vitro*, and *in vivo*, it can induce cell cycle arrest and apoptosis, as well as alter gene expression (Zhang et al., 2023). An antitumor protein isolated from the *Boletus edulis,* or in short BEAP, also exhibits similar antitumor effects (Zhang et al., 2021). BEP can inhibit the proliferation of breast cancer cells and induce apoptosis via mitochondrial pathways (Meng et al., 2021). Chen Zhao-li found that the ergost-type steroid compounds isolated from 95% ethanol extracts of *Phlebopus portentosus* exhibit notable cytotoxic activity gynecological tumor cells (Zhao-li et al., 2023). Iso-suillin, purified from the petroleum ether extract of *Suillus flavus* fruiting bodies, effectively induces apoptosis in human small cell lung cancer (SCLC) H446 cells (Zhao et al., 2016). It can also induce the death of K562 cells via the mitochondrial apoptotic pathway and the death receptor pathway (Wang et al., 2014). The 60% ethanol extracts of *Boletus auripes Peck*, *Leccinum crocip odiumWalt.*, *Boletus edulis Bull*, and *Boletus aereus* all demonstrate certain inhibitory effects on Caco-2 colon cancer cells. Among these, the inhibitory rate of the extract from *Leccinum crocip odiumWalt.* can reach 55.07% (Yu-yan, 2015). The polysaccharides extracted from *Boletus edulis* using the aqueous-alcohol precipitation method can extend the lifespan of S180 tumor-infected mice by 18.78% (Wei and Xincheng, 1999); a polysaccharide from the aqueous extract of *Boletus aereus* fruit (BAP) effectively inhibits the growth of S180 solid tumors and protects immune organs (Zheng et al., 2021). The BSF-A extracted from *Boletus speciosus Frost* achieves a 62.449% suppression rate against S180 tumors. BSF-A exhibits significant inhibitory effects on the growth of human laryngeal carcinoma Hep-2 and hepatoma HepG2 cells *in vitro*, with both dose and time dependency. Its mechanism of action primarily involves the activation of cell-mediated immune responses and direct cytotoxic effects (Hou et al., 2014). Table 1 summarizes the antitumor mechanisms of different *Boletus*.

**TABLE 1:** Mechanisms of antitumor effects in different Boletus species.

Species of boletes	Components	Tumor types	Antitumor mechanisms	References
*Boletus bicolor*	Protein D1, Protein-BEAP	A549 and H1299 non-small cell lung cancer (NSCLC) cells	Activates the MAPK signaling pathway; induces cell cycle arrest and apoptosis	Zhang et al. (2023), Zhang et al. (2021)
*Boletus edulis*	Polysaccharides extracted by hot water dissolution and alcohol precipitation	S-180 mouse ascitic tumor cells	Enhances host immune function; prolongs the lifespan of tumor-bearing mice	Wei and Xincheng (1999)
	Novel a cold-water-soluble polysaccharide (BEP)	MDA-MB-231 and Ca761 breast cancer cells	Inhibits cell proliferation; induces apoptosis via the mitochondrial pathway	Meng et al. (2021)
	Hydroethanolic extract of BE (obtained by ohmic heating green technology)	Caco-2 colorectal cancer cells	Induces G0/G1 phase cell cycle arrest, autophagy, and apoptosis	Quero et al. (2024)
*Phlebopus portentosus*	95% ethanol extract	HeLa cervical cancer cells, MCF-7 and MDA-MB-231 breast cancer cells (gynecological tumor cells)	Inhibits cell growth; exerts cytotoxic activity	Zhao-li et al. (2023)
*Suillus luteus*	Iso-suillin	BGC-823 human gastric cancer cells	Causes abnormal tumor cell metabolism; activates the apoptotic program	Yan-nan et al. (2022)
*Suillus flavus*	Iso-suillin	H446 human small cell lung cancer (SCLC) cells	Induces cell cycle arrest, inhibits tumor growth; triggers apoptosis via mitochondrial and death receptor pathways	Zhao et al. (2016)
		K562 human chronic myeloid leukemia cells	Induces G0/G1 phase cell cycle arrest; promotes K562 cell death via mitochondrial and death receptor pathways	Wang et al. (2014)
		A549 NSCLC cells	Induces early G1 phase cell cycle arrest and apoptosis	Yan Y. et al. (2021)
*Boletus auripes*	Ethanol-extracted polyphenolic compounds	Caco-2 colorectal cancer cells	Inhibits cell growth	Yu-yan (2015)
*Leccinum crocipodium*	-			
*Boletus aereus*	-			
*Boletus speciosus Frost*	Novel water-soluble polysaccharide (BSF-X, extracted via hot water extraction)	L929 mouse fibroblast cells (*in vitro*), S180 tumor cells (*in vivo*)	Inhibits tumor cell proliferation; promotes immune cell proliferation and function	Zhu et al. (2019)
	Novel water-soluble polysaccharide (BSF-A, extracted with 95% ethanol)	S-180 mouse ascitic tumor cells	Inhibits tumor growth; activates host immune response; induces tumor cell apoptosis	Hou et al. (2014)
		Hep-2 human laryngeal carcinoma cells	Inhibits tumor growth; activates host immune response; induces tumor cell apoptosis	
		HepG2 human hepatocellular carcinoma cells	induces tumor cell apoptosis (high concentration effective for HepG2 cells)	
*Leccinum rugosiceps*	Homogeneous water-soluble flaky polysaccharide (LRP-1, 18.82 kDa)	HepG2 human hepatocellular carcinoma cells, MCF-7 human breast cancer cells	Inhibits tumor growth; enhances host immune response	Li et al. (2020)

The multi-pathway antitumor effect of *Boletus* is mainly through its induction of apoptosis, blocking the cell cycle, and immune activation. This antitumor activity can be viewed as eliminating “cancer toxins (癌毒)” within the body, corresponding to the “clearing heat and removing toxins (清热解毒)” effect described in TCM. There may also be a potential connection between the TCM concept of “circulating blood and relaxing tendons (活血舒筋)” and the influence on the tumor microenvironment through improved microcirculation. Current research primarily focused on *in vitro* cellular experiments and limited animal models. There is a lack of systematic preclinical pharmacodynamic and toxicological evaluations, indicating that clinical application is still distant. Future efforts should emphasize a deeper investigation into the antitumor structure-activity relationships of its active components, the metabolic processes *in vivo*, and target specificity.

### 3.4 Cardiovascular protection

*Boletus* also demonstrates significant medicinal value in cardiovascular protection. *Boletus* polysaccharides exhibit lipid-lowering and hypoglycemic effects in streptozotocin (STZ)-induced type 2 diabetes mellitus (T2DM) mice, manifested by reductions in fasting blood glucose (GLU), malondialdehyde (MDA), and triglyceride (TG) levels. *Boletus* edulis extracts can lower blood glucose levels in T2D mice and modulate the microbiota, normalizing its pattern (Zanfirescu et al., 2023). *Boletus* *edulis* extract has also been reported to modulate dysbiotic microbiota (Avram et al., 2023). The dietary fiber and unsaturated fatty acids abundant in *Boletus* contribute to lowering cholesterol and TG levels in the blood, thereby preventing the onset of atherosclerosis (Threapleton et al., 2013). Oral hot water extracts from *Boletus* for 18 consecutive weeks can effectively reduce the blood pressure and heart rate in spontaneously hypertensive rats (SHR). The reduction of blood urea nitrogen (BUN), creatinine (Cre), and TG, as well as an increase in high-density lipoprotein cholesterol (HDL-C) (Midoh et al., 2013). *Boletus aereus* polysaccharide-b (BAP-b) can regulate macrophages to secrete cytokines via the TLR4/NF-κB pathway, thereby regulating the blood lipid metabolism of obese mice (Chun-Xia, 2024).

The phenotypic transformation of vascular smooth muscle cells (VSMCs) is the common pathophysiological basis for vascular reconstruction diseases such as hypertension and atherosclerosis. In the physiological state, VSMCs present a highly differentiated contraction state, which can control and maintain the tension and elasticity of blood vessels. In the pathological state, the VSMCs in the damaged blood vessels are dedifferentiated from contractile to synthetic, which will lead to disorders in the structure and function of blood vessels and the enhancement of inflammatory reactions. Thus, curbing VSMC phenotypic transformation is crucial for averting vascular injury-related diseases (Bkaily et al., 2021). The decrease in the expression of actin-related protein smooth muscle 22 alpha (SM22α) is a specific sign of the dedifferentiation of VSMCs. Iso-Suillin hinders the phosphorylation of SM22α through the targeted PKCδ signaling pathway, causing the cell cycle to stagnate in the G0/G1 phase, thus inhibiting proliferation and finally preventing the phenotypic transformation of VSMCs. Iso-Suillin has great potential in the treatment of vascular remodeling diseases (Pin et al., 2017).

The inhibition of angiotensin-converting enzyme (ACE) activity is one of the effective strategies for treating hypertension. KYPHVF (KF6), a hexapeptide isolated from *Boletus griseus*, has an ACE inhibitory activity (IC_50_ = 5.99 μmol/L). In spontaneously hypertensive rats, oral administration of KF6 (10 mg/kg for 5 weeks) lowered blood pressure, reduced RAAS-related factors (ACE, AGT, ALD, and ANG II), and alleviated cardiac and renal fibrosis, inflammation, and oxidative stress, with effects comparable to those of the conventional ACE inhibitor captopril (10 mg/kg). Moreover, KF6 uniquely improved gut microbiota composition by increasing SCFA-producing bacteria (Huan et al., 2025). Due to its strong biological activity, KF6 is expected to lower blood pressure and reduce the damage of hypertension to heart and kidney tissue.

In addition, the potassium in *Boletus* helps to promote sodium excretion and maintain electrolyte balance. These protective effects on the heart show that *Boletus* may be of great value in the prevention and management of cardiovascular problems.

### 3.5 Toxicity

Although *Boletus* is highly respected for its edible and medicinal value, some species contain natural toxins, which can lead to poisoning if eaten accidently. Therefore, addressing toxicity risks must be a priority, especially when applied in healthcare, agriculture, and other sectors, in order to safely expand its use. The clinical symptoms of *Boletus* poisoning are usually gastroenteritis, neuropsychiatric, or mixed types. While fatal toxins are rare, gastroenteritis is the most common presentation, characterized by nausea, vomiting, abdominal pain, and diarrhea (Jifen, 2015; Zhang Y. et al., 2025). Given the differences in toxic components among various poisonous *Boletus* species, the clinical manifestations also vary. Toxins such as muscarine and bufotenin can cause psychotic symptoms, including excitement, confusion, abnormal behavior, visual hallucinations, and auditory hallucinations. What’s more, psilocybin induces micropsia (Xiao-wei et al., 2010). *Suillus* and *Tylopilus*, genera within the *Boletaceae* that exhibit gastrointestinal poisoning, while some *Boletus* species (e.g., *Boletus speciosus*) cause neurological symptoms. It has been found that very toxic species, such as *Butyriboletus roseoflavus* and *Boletus satanas*, can cause liver damage and hemolysis (Niankai and Quanying, 2024). Crucially, a large number of dangerous *Boletus* species are regarded as “conditionally edible,” which means that with the right preparation, they can be made safe for ingestion. Techniques such as high-temperature boiling or slicing followed by blanching effectively inactivate many of their toxins. Nonetheless, some harmful substances, such as palilline, are resistant to remove by conventional cooking methods due to their heat persistence (Wennig et al., 2020).

Current knowledge gaps concerning *Boletus* toxicity primarily relate to the chemical nature of toxins, ambiguous species identification, and a lack of epidemiological data. Many toxic species remain unisolated and unidentified, with their target sites and metabolic pathways unknown; precise identification of venomous species requires integrating genomics and metabolomics; clinical poisoning cases lack standardized toxin identification and long-term follow-up. Future research should focus on toxin isolation, purification, toxicological evaluations, and clinical investigations of poisoning epidemiology.

## 4 Medicinal value from the perspective of TCM

Unlike modern pharmacology, which tends to focus on single active components, TCM emphasizes a holistic, dynamic, and pattern-based approach to health and disease. It emphasizes that human body is an organic whole centered around the five zang organs (identified as the heart, liver, spleen, lungs, and kidneys, which are central to TCM physiology), and that all substances, including the human body itself, is in constant motion. The essence of disease lies in imbalances of Yin and Yang (defined as opposing but complementary qualities whose balance maintains health), dysfunction of zang-fu (the internal organs of human), and disturbances in the flow of Qi (the intangible, high-mobility nutritive substance that maintains vital activitie), blood, and bodily fluids (thinner part of fluids (jin) and thicker part of liquid (ye)).

In this framework, the therapeutic properties of Chinese herbal medicines are derived from long-term observation of their effects on the body, and their efficacy often arises from the combined action of multiple bioactive constituents. The characteristic of “nature, flavor, and meridian tropism (性味归经)” of Chinese herbal medicine reflects this overall treatment method. The meridians and collaterals(经络) are the channels through which qi and blood flow throughout the body, connecting organs and limbs, connecting the upper and lower parts, inside and outside of the body. They form a functionally regulated network system.

In classical TCM records, *Boletus* is described as having a mild sour and pungent flavor, a neutral property, and an affinity for the heart and spleen meridians. From a modern scientific perspective, the sour taste comes from its richness in soluble sugars, organic acids, and free amino acids, while the pungent flavor originates from various volatile compounds. In TCM theory, sour flavor has astringent and consolidating effects, while pungent flavor promotes qi circulation, dispersion, and blood flow. Its neutral property balances yin and yang within the body. The heart governs the blood and vessels (心主血脉), promoting blood circulation to nourish the entire body, and also governs the bright spirit (主神志). The spleen governs transportation and transformation (脾主运化) and ascends the nutrients (主升清), ascending to manage the digestion, absorption, and distribution of nutrients from food and drink. It serves as the source of qi and blood generation (气血生化之源) and contains blood (主统血). The coordinated function of the heart and spleen jointly maintains the fullness and flow of qi and blood. The characteristic of *Boletus* to enter both the heart and spleen meridians enables it to coordinate the functions of these two core organs, subsequently impacting the generation and circulation of qi and blood as well as stabilizing mental functions. This forms the material basis and mechanism of funtion for its traditional efficacy described below.

### 4.1 Clearing heat and relieving vexation (清热解烦)

*Dian Nan Ben Cao* records that *Boletus* can clear heat and relieve vexation, indicating its capability to harmonize yin and yang, treating heat-related disorders and alleviating the body’s suffering from pathogenic heat (Mao and Guo-You, 2013). When there is an invasion of external pathogenic heat or excessive yang damaging yin, the internal yang becomes overly excessive, showing clinical symptoms such as fever with a preference for coolness, irritability, thirst with dry throat, constipation, and scanty dark urine-symptoms indicative of “interior heat pattern (实热证)”. The heart governs fire (心主火), and *Boletus* enter the heart meridian, so it can clear “pathogenic heat (热邪)” from the body, balance yin and yang, eliminate excess pathogenic heat, and alleviate restlessness.

From a modern scientific standpoint, this efficacy is mainly related to its anti-inflammatory and antioxidant properties. In particular, “clearing heat” can be understood as reducing inflammatory responses and oxidative stress, while “relieving vexation” may correspond to alleviating inflammation-related stress states and immune dysregulation. *Boletus* polysaccharides can enhance anti-inflammatory effects while reducing pro-inflammatory responses in asthma mouse models. Its efficacy is closely related to the Th1/Th2 cells balance, mainly by promoting the production of Th1 cytokines while inhibiting the excessive secretion of Th2 cytokine, to reestablish immune balance and boost body’s anti-infection ability, which is consistent with the idea of “spontaneous harmonization between yin and yang (阴阳自和)” in TCM principle (Wu et al., 2016).

The abundant antioxidant components in *Boletus* exert strong antioxidant effects, inhibiting inflammatory mediators through pathways like NF-κB and regulating cell proliferation, differentiation, and apoptosis via MAPK signaling pathway (Zhang et al., 2020; Zhang et al., 2023). Furthermore, Wei et al. discovered that a novel water-soluble *Boletus* polysaccharide can lower cytokine levels by activating the MANF-BATF2 signaling pathway, and remodel gut microbiota, improving inflammatory bowel disease (IBD) (Wei et al., 2024).

### 4.2 Nourishing blood and tonifying Qi (补血益气)

*Dian Nan Ben Cao* mentions that *Boletus* polysaccharides possess the efficacy of “nourishing blood and tonifying qi” (Mao and Guo-You, 2013). “Nourishing blood” is traditionally associated with supporting blood-related vitality and nutritional status, and may be loosely linked to erythropoiesis and hemoglobin synthesis, while “tonifying qi” enhances energy metabolism and improves endurance and resistance to fatigue. In TCM, “qi is the commander of the blood (气为血之帅),” meaning qi drives blood circulation. From a modern scientific perspective, this concept may be loosely related to cardiovascular dynamics, microcirculatory perfusion, and metabolic energy support.

The concept of Ying-nutrient qi (营气)—the nutritive qi that flows within blood vessels—can be understood in modern terms as the integrated functions of hematopoiesis, plasma nutrient composition, and oxygen transport capacity. *Boletus* may support this interpretation through its nutritional composition, including proteins, minerals, and B vitamins. For instance, *Suillus bovinus* contains iron levels exceeding 1000 mg/kg—far higher than common cultivated mushrooms such as king oyster and lion’s mane, which are directly utilized for hemoglobin synthesis and improving oxygen-carrying capacity (Ya-Yuan et al., 2022). Moreover, the high protein and B-vitamin content in *Boletus* can stimulate bone-marrow hematopoietic stem cells, thus speeding the synthesis and maturation of red blood cells (Jia et al., 2007). As a result, *Boletus* may contribute to blood nourishment and provide nutritional support relevant to blood maintenance, although direct evidence for a specific anti-anemic effect remains limited.

Furthermore, its immunomodulatory characteristics enhance the body’s immunity and resistance, boosting the “Wei-defensive qi (卫气)” —the protective qi that travels outside the vessels, which aligns with modern immunology. The Wei-defensive qi safeguards the body against external pathogens (外邪)—disease-causing elements that invade the human body from the external environment in TCM theory, including wind, cold, heat, dampness, dryness, and fire. The immunomodulatory effects of *Boletus*, particularly its polysaccharides, directly strengthen Wei-defensive qi. For example, BSF-X and BSF-A from *Boletus* speciosus activate macrophages and T lymphocytes, upregulate pro-inflammatory cytokines (IL-6, TNF-α), and increase immune organ weights (Zhu et al., 2019; Hou et al., 2014). BEP promotes the transformation of spleen lymphocytes and boosts white blood cell counts (Wei and Xincheng, 1999). These effects may be viewed as consistent with the traditional function attributed to Wei-defensive qi in TCM.

In summary, the TCM description of “nourishing blood and tonifying qi” is not merely a metaphorical statement but finds concrete scientific grounding in the hematopoietic (iron, B vitamins), hemodynamic (polysaccharides, ACE-inhibitory peptides, potassium), and immunomodulatory (polysaccharide-mediated immune cell activation) actions of *Boletus*. These effects may be viewed as consistent with the traditional function attributed to Wei-defensive qi in TCM.

### 4.3 Circulating blood and relaxing tendons (活血舒筋)

The phrase “circulating blood and relaxing tendons” refers to promoting blood flow and the relaxation of tendons and meridians. The most common cases include contusions and joint pain. From a modern perspective, this principle emphasizes reducing pain and swelling through anti-inflammatory mechanisms and promoting blood circulation to alleviate congestion and oxidative stress-related injuries (Hongjun et al., 2023). This therapeutic principle is primarily applied to treat conditions caused by qi and blood stasis and inadequate nourishment of tendons and meridians. It also reduces oxidative stress damage to the body and accelerates the repair of injured tissues by promoting extracellular matrix metabolism. Moreover, it supports extracellular matrix metabolism, facilitating tissue repair.

*Chinese Materia Medica* suggests *Boletus* can dispel wind and dissipate cold, relax tendons and soothe meridians, treating lower back pain and leg pain, limb numbness, and meridian blockages (Zhu, 2024). Zhang et al. validated that *Boletus* may alleviate muscle atrophy induced by lipopolysaccharide (LPS) via the PI3K/AKT signaling pathway (Zhang M. et al., 2025). *Boletus* is one of the herbal ingredients in TCM formulations “Shujin Pills”, which is for muscle relaxation. This effect may partially explain the traditional TCM function of “relaxing tendons and soothing meridians”, as preservation of muscle mass and function contributes to improved mobility and relief of musculoskeletal discomfort.

The mildly acidic nature of *Boletus* aligns with the “sour enters the liver (酸入肝)” notion in five-element theory—sour foods nourish the liver, maintaining liver yin and blood, which in turn supports the tendons. The liver governs the free flow of qi (肝主疏泄), meaning that the liver maintains free flow of qi over the entire body. TCM holds that tendons are governed by the liver. Tendons attach to bones and joints, enabling muscle and joint movement. Sufficient liver blood flow helps to keep tendons strong and joints flexible, thus improving overall endurance. When the liver perfusion is insufficient, the tendons and meridians may not get enough nutrition, thus reducing physical performance. Therefore, *Boletus* cloud enhances the smoothness of meridian circulation, strengthens tendons and bones, and relieves the symptoms associated with qi and blood stagnation (such as stiffness and joint pain). These benefits show that it has a therapeutic effect on diseases such as rheumatic pain, arthritis, and limb numbness.

Recent studies have shown that *Boletus* extract can reduce cholesterol and TG, and purified polysaccharides and peptides also exhibit good lipid-regulating effects (Chun-Xia, 2024; Huan et al., 2025; Threapleton et al., 2013). In addition, a variety of nutrients in *Boletus* support blood overflow and contribute to its “blood-circulating” characteristics. For example, polysaccharides enhance the blood flow and lower its viscosity, while vitamins and minerals shield vascular walls, maintaining their integrity and preventing cardiovascular issues like atherosclerosis. This alleviates problems caused by poor circulation, such as cold hands and feet, pale complexion, etc., so that the tendons and bones can function normally.

### 4.4 Dispersing wind and eliminating dampness (祛风除湿)

The concept of “dispersing wind and eliminating dampness” primarily manifests through its anti-inflammatory, antioxidant, and immunomodulatory effects, as well as by regulating the extracellular matrix to increase resilience against diseases (Cen et al., 2023). There is a saying in TCM that “unobstructed flow leads to no pain”. By reducing rheumatic accumulation and unblocking meridians, symptoms such as discomfort and numbness caused by obstructions can be alleviated. *Boletus* may inhibit the activity of rheumatic factors on joints and muscles, and prevent the rheumatic accumulation of poisons in the body.

Phenolic compounds in *Boletus edulis* have been confirmed to have significant anti-inflammatory and cartilage protection properties in osteoarthritis (OA) models by greatly reducing inflammatory mediator expression and inhibiting NF-κB p65 nuclear translocation (Paesa et al., 2025). Phenolic compounds and polysaccharides from *Boletus* edulis have been shown to exert significant anti-inflammatory, immunomodulatory, and antioxidant effects, including reductions in pro-inflammatory cytokines, inhibition of NF-κB p65 nuclear translocation, and protection of cartilage in OA models (Paesa et al., 2025). These pharmacological activities correspond to key pathological processes in rheumatic and musculoskeletal disorders and may provide a modern scientific basis for the traditional TCM concept of “dispersing wind and eliminating dampness”, which is thought to prevent the accumulation of “rheumatic toxins” and relieve joint and muscle pain. Therefore, *Boletus* may play a supportive therapeutic role in such conditions, although further systematic studies are needed for confirmation. This effect of removing wind and transforming dampness is to treat joint pain and muscle pain caused by excessive dampness in the body, and also enhance the overall conditions of patients with rheumatic diseases. Therefore, it can be considered that *Boletus* may play a certain auxiliary therapeutic role for patients with rheumatic arthritis and similar conditions, but further systematic studies are needed for confirmation.

## 5 Perspective and applications in pharmacology

The ongoing research on the medicinal properties of *Boletus* needs to go beyond the limitations of the method of focusing on a single component and solve the complex nature of the disease. The former failed to fully capture the overall effectiveness of *Boletus*, and there were defects in bioavailability and targeting. The latter is attributed to the limited influence of the targeted single component. Therefore, the utilization of *Boletus* must break away from the traditional pharmacological model of “single component-single target,” and deeply study the inherent “self-synergistic” mechanism and the potential for “compatibility with external herbs,” so as to develop compound formulas that enhance efficacy and minimize toxicity, and use network pharmacology to reveal the medicinal potential.

In this review, self-synergy refers to the complementary interactions among multiple bioactive constituents within *Boletus*, including polysaccharides, phenolic compounds, sterols, and other metabolites, which may collectively contribute to its pharmacological activities through multi-component and multi-target regulation. Mutual synergy refers to the enhanced therapeutic potential achieved through the compatibility of *Boletus* with other medicinal materials in traditional formulations. This concept may provide a useful framework for future research and drug development by emphasizing the evaluation of multi-component interactions rather than isolated compounds alone (Wang C. et al., 2026; Wang X. et al., 2026). For example, polysaccharides are primarily associated with immunomodulatory activities, whereas phenolic compounds contribute substantially to anti-inflammatory and antioxidant effects, and their complementary actions may jointly contribute to the medicinal value of *Boletus*.

Despite the growing body of evidence supporting the pharmacological activities of *Boletus,* most current studies are limited to *in vitro* experiments, animal models, or computational predictions, while high-quality clinical evidence remains lacking. Furthermore, the active constituents responsible for specific therapeutic effects and their interactions have not been fully elucidated, highlighting the need for further mechanistic and translational studies.

### 5.1 Deepening self-synergistic mechanisms

At the basic research level, proteomics and metabolomics techniques can be used to describe the interaction networks of polysaccharides, flavonoids, steroids, terpenes and peptides in *Boletus*. This description helps to determine which metabolites contribute to specific biological activities and determine their optimal relative concentrations. Further research on these molecular interactions clarifies how they affect absorption, distribution, metabolism, and targeting efficiency in the body. The antioxidant activity of *Boletus* provides a clear illustration of self-synergy. Polysaccharides scavenge free radicals directly, flavonoids chelate pro-oxidant metal ions, and steroidal compounds activate endogenous antioxidant enzymes. When these metabolites are present together in a crude extract, their complementary activities may contribute to stronger overall biological effects than those observed for individual purified compounds, suggesting that part of the biological activity may depend on interactions among multiple metabolites. This observation supports the use of whole extracts or standardized multi-component fractions rather than purified single compounds for therapeutic applications. In addition, the insights obtained from the “self-synergy” research can provide information for the development of standardized, fingerprint-controlled extracts or combination formulas from different species of *Boletus*. This method promotes the creation of functional foods, dietary supplements or supportive therapeutic products by taking advantage of natural ingredients, thus yielding more consistent, comprehensive bioactive preparations.

### 5.2 Exploring compatibility with external herbs

Utilizing network pharmacology and integrating TCM’s “monarch-minister-assistant-guide (君臣佐使)” formula principles and “nature, flavor and meridian tropism (性味归经)” theory, to identify synergistic partner herbs that enhance the efficacy of *Boletus* or address co-existing conditions is key to achieving precise application of *Boletus* and harnessing the advantages of TCM compound formulas. For instance, when *Boletus* serves as the monarch medicine for effects such as “clearing heat and relieving vexation” or “nourishing blood and tonifying qi”, the minister medicines should enhance these actions or address concomitant symptoms. Examples include astragalus enhancing qi tonification and immune boosting, honeysuckle clearing heat and detoxifying, salvia promoting blood circulation, and hawthorn invigorating blood and dispersing stagnation. Assistant medicines should be selected to assist the monarch and minister medicines in treating secondary symptoms, eliminate or mitigate the toxicity or potency of the monarch and minister medicines, or counteract adverse effects. Guide medicines should be chosen to direct the action of the formula to the affected area and harmonize the actions of all herbs.

### 5.3 Network pharmacology-driven strategies

Utilizing databases such as the Traditional Chinese Medicine Systems Pharmacology Database (TCMSP), PubChem, and SwissTargetPrediction to predict the important bioactive metabolites of *Boletus* and their potential targets is a practical strategy for clarifying its pharmacological basis. This approach is particularly relevant because *Boletus* contains multiple bioactive metabolites that may act synergistically rather than through a single target. In fact, Zhang M. et al. (2025) successfully integrated whole-plant targeted metabolomics, network pharmacology, molecular docking, and molecular dynamics to explore the effects of *Boletus* edulis on muscle atrophy, identifying sesamin as a key active compound and suggesting the PI3K/AKT signaling pathway as a major mechanism.

Employing resources like the Kyoto Encyclopedia of Genes and Genomes (KEGG) and Gene Ontology (GO) for pathway enrichment analyses can reveal shared pathways related to diseases. Building a multi-dimensional network of “drugs-components-targets-pathways-disease” and analyzing the tonic structure of the network, which helps identify the key targets that are jointly regulated by *Boletus* and compatible herbs. Understanding complementary pathways facilitates comprehensive modulation. Furthermore, predicting potential toxic targets allows for the selection of compatible herbs that alleviate toxicity, thereby optimizing the role of adjunctive botanicals. Based on these analyses, future studies can prioritize and rank potential compatibility combinations to identify the most synergistic pairs and compound mixtures.

### 5.4 Application prospects

Based on the above theoretical analysis and network predictions, it is possible to design and develop innovative TCM formulations with *Boletus* as either a monarch or minister ingredient, which can be used for specific indications such as chronic inflammatory disease management and as adjuncts in cancer therapies. Various end products such as standardized extracts, capsules, tablets, and functional foods can be produced. If we want to further achieve precise health interventions, we can explain individual differences from the theoretical level of TCM, that is, different constitutions and syndromes, and provide personalized strategies for different groups of people. For example, people with qi deficiency can improve their energy level by mixing *Boletus* with astragalus, while people with qi stagnation can mix *Boletus* with salvia or hawthorn. Similarly, from the perspective of modern medicine, exploring how to combine *Boletus* with the established formula of qi and blood can improve the therapeutic effect and help clarify their synergy.

## 6 Conclusion

Modern medical philosophy takes prevention as the core, which makes the ideas of “prevention-and-treatment integration (防治结合)” and “phytogenic-pharmaceutical homology (药食同源)” of TCM attract attention again. As a valuable natural resource, the therapeutic potential of *Boletus* is the main goal of current scientific research. As a valuable natural resource, the therapeutic potential of *Boletus* has attracted increasing scientific attention. Pharmacological studies have demonstrated that *Boletus* exhibits diverse biological activities, including antioxidant, antitumor, immunomodulatory, and metabolic regulatory effects. These findings are generally consistent with the traditional TCM view that *Boletus* species may clear heat, nourish blood, dispel wind, and relax tendons. These mechanisms involve multiple components, targets, and pathways, showing the interaction of synergy. Core antioxidant and anti-inflammatory activities act as defensive shields, and immunomodulation enhances the body’s defenses and supports processes such as apoptosis and cell-cycle block, which support its antitumor properties. In addition, the impact of *Boletus* on metabolism and cardiovascular function suggests its ability to prevent and manage chronic diseases. This comprehensive mode of action is in line with the principles of TCM and supports overall balance, coordinated organ cooperation, and the intrinsic connection between drugs and food.

A comprehensive assessment of the medicinal potential of *Boletus* requires the combination of TCM insights with contemporary scientific tools. This interdisciplinary strategy transcends the limitations of single compound research and makes full use of its natural “self-synergistic” characteristics. Within the framework of TCM, it is very important to explore how *Boletus* can be effectively combined with other herbs. These studies should follow the key principles of TCM of “preventing a disease before it arises and preventing transmission of a disease after its onset (未病先防,既病防变)”, and at the same time strive to build a more comprehensive treatment plan and promote the innovation of TCM.
